# Interleukin-17A Promotes MUC5AC Expression and Goblet Cell Hyperplasia in Nasal Polyps via the Act1-Mediated Pathway

**DOI:** 10.1371/journal.pone.0098915

**Published:** 2014-06-03

**Authors:** Wentong Xia, Jing Bai, Xingmei Wu, Yi Wei, Shaoyan Feng, Lei Li, Jia Zhang, Guanxia Xiong, Yunping Fan, Jianbo Shi, Huabin Li

**Affiliations:** 1 Allergy and Cancer Center, Otorhinolarygology Hospital, The First Affiliated Hospital of Sun Yat-sen University, Guangzhou, China; 2 Department of Otolaryngology, Head and Neck Surgery, Xinhua Hospital, Shanghai Jiaotong University School of Medicine, Shanghai, China; 3 Department of Otolaryngology, The Fifth Affiliated Hospital of Sun Yat-sen University, Zhuhai, China; University of Nebraska Medical Center, United States of America

## Abstract

**Background:**

Recent studies demonstrated that nasal polyps (NP) patients in China and other Asian regions possessed distinct Th17-dominant inflammation and enhanced tissue remodeling. However, the mechanism underlying these observations is not fully understood. This study sought to evaluate the association of interleukin (IL)-17A with MUC5AC expression and goblet cell hyperplasia in Chinese NP patients and to characterize the signaling pathway underlying IL-17A-induced MUC5AC expression *in vitro*.

**Method:**

We enrolled 25 NP patients and 22 normal controls and examined the expression of IL-17A, MUC5AC and act1 in polyp tissues by immunohistochemical (IHC) staining, quantitative polymerase chain reaction (qPCR) and western blot. Moreover, by using an *in vitro* culture system of polyp epithelial cells (PECs), IL-17A-induced gene expression was screened in cultured PECs by DNA microarray. The expression of IL-17RA, IL-17RC, act1 and MUC5AC and the activation of the MAPK pathway (ERK, p38 and JNK), were further examined in cultured PECs and NCI-H292 cells by qPCR and western blotting, respectively.

**Results:**

We found that increased IL-17A production was significantly correlated with MUC5AC and act1 expression and goblet cell hyperplasia in polyp tissues (*p*<0.05). IL-17A significantly stimulated the expression of IL-17RA, IL-17RC, act1 and MUC5AC, and the activation of the MAPK pathway in cultured PECs and NCI-H292 cells (*p*<0.05). In addition, IL-17RA, IL-17RC and act1 siRNA significantly blocked IL-17A-induced MUC5AC production *in vitro* (*p*<0.05).

**Conclusion:**

Our results suggest that IL-17A plays a crucial role in stimulating the production of MUC5AC and goblet cell hyperplasia through the act1-mediated signaling pathway and may suggest a promising strategy for the management of Th17-dominant NP patients.

## Introduction

Nasal polyps (NP) is characterized by specific Th2-skewed, eosinophilic inflammation and extensive edema in polyp tissues [1]. Th2-dominant eosinophilic inflammation and subsequent tissue edema are the key features of western NP patients, as demonstrated by marked infiltration of activated eosinophils, a high extent of tissue edema and paucity of submucosal glands [2]. Of interest, some recent studies have demonstrated that NP patients in China and other Asian regions had distinct inflammatory patterns compared with their Caucasian counterparts [3]–[5]. For example, Kim et al. reported that non-eosinophilic NP patients comprised 66.7% of the total Korean patients included in their study [3]. Cao et al. reported more than half of Chinese NP patients presented a Th17-dominant, neutrophilic inflammation pattern [4]. In the recent study by Shi et al., they found significantly enhanced mucous metaplasia and tissue remodeling in non-eosinophilic NP patients in China [5]. However, the underlying mechanism is not yet fully understood.

Th17 cells are newly emerged immune/inflammatory cell subsets which are now widely believed to be critical for the regulation of various chronic immune diseases [6]. As the characteristic cytokine of Th17 cells, IL-17A has been revealed to play a significant role in regulating inflammation, modulating airway structural cells and stimulating innate immunity to mediate neutrophil recruitment in asthma and other airway diseases [7]. Investigations have revealed that IL-17A can orchestrate local inflammation by inducing the release of proinflammatory cytokines, such as TNF-α, IL-1β, G-CSF and IL-6, as well as the chemokines CXCL2 and IL-8, produced by human bronchial fibroblasts, epithelial cells and airway smooth muscle cells [8]–[10]. Furthermore, IL-17A can act in synergy with IL-6 to induce the expression of the mucus proteins MUCAC and MUC5B [11]. On the other hand, mucin overproduction is one of the hallmarks of chronic airway diseases such as chronic obstructive pulmonary disease, asthma and NP [12]. Excessive mucus production can increase morbidity and mortality by disturbing proper mucociliary and innate immune functions in the airway. Of the several secretory mucin genes, MUC5AC is particularly considered to be a marker of mucus metaplasia because of its high expression in mucus-secreting goblet cells [13]. Therefore, targeting MUC5AC regulation may represent a promising strategy for NP treatment [12], [14].

To date, numerous reports have shown that both Th2 and Th17 cells are able to prominently stimulate MUC5AC gene expression in airway epithelial cells either *in vitro* or *in vivo* [14]–[16]. However, whether and how Th17 promotes MUC5AC production and goblet cell hyperplasia in NP patients remains unclear.

## Materials and Methods

### Ethics statement

The research protocols were approved by the Ethics Committee of the First Affiliated Hospital of Sun Yat-sen University. All NP and control participants with tissue examination provided their written informed consent to participate in this study.

### Patients and tissue samples

Adult NP subjects (n = 25) were recruited from the First Affiliated Hospital of Sun Yat-sen University. The diagnosis of NP was made according to the current European Position Paper on rhinosinusitis and nasal polyps [1]. All patients had undergone an unsuccessful standardized course of medical therapy (oral and/or nasal glucocorticoid, antibiotics, antileukotrienes and nasal irrigation for more than 12 weeks) and consented to tissue collection at the time of surgery. None of the subjects used oral or nasal glucocorticoids or other medications (e.g., antibiotics or antileukotrienes) for 4 weeks before sample collection. During endoscopic surgery, the polyp tissues were sampled for experimental use. As normal controls, 22 patients undergoing septoplasty due to anatomical variations were enrolled, and the tissues from the inferior turbinate were sampled during septal surgery. The demographic data of all subjects enrolled in this study are listed in Table S1 in File SI.

Tissue samples were divided into 3 portions: the first portion was frozen in liquid nitrogen and stored at −80°C for subsequent RNA isolation, the second portion was used for protein isolation, and the third portion was fixed overnight in a freshly prepared fixative containing 4% paraformaldehyde in PBS (pH 7.4) and was embedded in paraffin wax for histological staining. In addition, some fresh polyp tissues were further used for the isolation and culture of polyp epithelial cells (PECs) to assess the effect of IL-17A on MUC5AC expression *in vitro*.

### Histological staining

Paraffin sections (4 µm) were used for histological staining. PAS staining was performed to evaluate goblet cell hyperplasia. IHC staining was performed by the peroxidase-labeled streptavidin-biotin technique as we described elsewhere. The sections were incubated overnight at 4°C in the presence of anti-IL-17A (R&D systems, Minneapolis, MN, USA) and anti-MUC5AC antibodies (Santa Cruz, Santa Cruz Biotech, CA, USA). Thereafter, each section was incubated with a secondary antibody and then with horseradish peroxidase-labeled streptavidin complex (Zhongshanjinqiao, Beijing, China). The distribution of peroxidase was revealed by incubating the sections in a solution containing 3% 3,3-diaminobenzidine tetrahydrochloride before being counterstained with hematoxylin and coverslipped. Negative control studies were performed by replacing the primary antibodies with normal IgG in appropriate concentrations.

The PAS staining and MUC5AC staining were semi-quantitatively scored on a scale of 0 to 4 (0, no staining; 1, mild; 2, medium; 3, severe; 4, extremely severe) in 10 randomly selected HPFs (×400 magnification) and then averaged. The number of IL-17A^+^ cells was determined by counting the positive-staining cells in 10 randomly selected HPFs (×400 magnification) and then averaged.

### Real-time quantitative polymerase chain reaction (qPCR)

The mRNA expression levels of IL-17A, IL-17RA, IL-17RC, MUC5AC and act1 were detected by real-time qPCR analysis. Briefly, total RNA was extracted with TRIzol reagent (Invitrogen, Carlsbad, CA, USA) following the manufacturer's instructions. Reverse transcription was performed, and the cDNA was synthesized from 2 µg of total RNA using an oligo (dT)18 primer and M-MLV reverse transcriptase (TAKARA, Syuzou, Shiga, Japan) for quantitative PCR. RNA integrity and the success of the reverse transcription reaction were monitored by PCR amplification of glyceraldehyde-3-phosphate dehydrogenase (GAPDH) transcripts. Expression of mRNA was determined using the ABI PRISM 7600 Detection System (Applied Biosystems, Foster City, CA, USA) and SYBR Premix (TAKARA). The primer sequences of each gene used in the study are listed in Table S2 in File SI. PRISM samples contained 1×SYBR Green Master Mix, 1.5 µL of 5 µM primers, and 25 ng synthesized cDNA in a 25-µL volume. Reactions were heated to 95°C for 10 min followed by 40 cycles of denaturation at 95°C for 10 s and annealing and extension at 60°C for 60 s. Melting curve analysis was used to control for amplification specificity. The mean threshold cycle (Ct) values were normalized to GAPDH, and the relative mRNA levels of target genes were analyzed with the 2^−ΔΔCt^ method. Experiments were performed in triplicate for each data point.

### Western blot analysis

The protein levels of act1, total p38, ERK, JNK and phosphorylated p38, ERK and JNK (p-p38, p-ERK and p-JNK) were detected by western blot analysis. Briefly, the tissues or cells were collected, and total protein was extracted in 100 µL of RIPA lysis buffer at 4°C for 30 min. The protein concentration was determined by the Bradford method. Samples containing 10 µg of protein were boiled, subjected to SDS-PAGE in 10% Tris-glycine gels and transferred electrophoretically to a polyvinylidene fluoride membrane. The membrane was incubated with 5% fat-free milk in Tris-buffered solution (TBS) containing 0.05% Tween 20 (1 h, room temperature) and then incubated with primary antibodies (against total p38, ERK, JNK, p-p38, p-ERK, p-JNK and GAPDH, each diluted 1∶1000 to 2000; all were purchased from Cell Signaling, Danvers, MA, USA; anti-act1, 1∶1500; Genetex, Irvine, CA, USA) overnight at 4°C. The membrane was then incubated with horseradish peroxidase-linked secondary antibody and finally processed using the ECL chemiluminescence reaction kit (Cell Signaling), followed by exposure on medical film. The relative band density of the target protein compared with GAPDH was quantified with the Bio-Rad Quantity One 1-D Analysis Software (Bio-Rad, Hercules, CA, USA).

### Enzyme-linked immunosorbent assay (ELISA)

The level of secreted MUC5AC in the supernatants of cultured cells was measured by using a MUC5AC sandwich ELISA developed in our laboratory with anti-MUC5AC antibody (Santa Cruz). Briefly, 10 µL of collected sample and 40 µL of diluent were added to 96-well plates pre-coated with captured MUC5AC antibody (1∶200) for assay. After incubation and washing, anti-MUC5AC antibody (1∶200) was added into each well. After incubation and washing again, 100 µL of horseradish peroxidase-goat anti-mouse IgG conjugate (1∶10,000) was dispensed into each well. The color reaction was developed with 3,3′, 5,5′-tetramethylbenzidine peroxide solution and stopped with 1 M H_2_SO_4_. The absorbance was read at 450 nm using a microplate reader (Bio-Rad), and the optical density (OD) value at 450 nm was recorded.

### Cell culture, stimulation and siRNA interference

For *in vitro* cell culture, primary PECs were randomly collected from 5 NP patients by means of enzymatic digestion. Collected cells were rinsed in 5 mL Dulbecco's modified Eagle's medium/F12, then transferred into BEGM medium (Lonza, Walkersville, MD, USA) and poured into a plastic flask for overnight incubation at 37°C in a 5% CO_2_ atmosphere. The PECs were collected after 5–7 days. Next, PECs and NCI-H292 cells (purchased from ATCC, MD, USA) were cultured in submersion cultures in BEGM medium (Lonza, Walkersville, MD, USA) until passaged. When 80–90% confluence was reached, the epithelial cells were washed with PBS (37°C, pH 7.4), and fresh medium without hydrocortisone was added in the presence of recombinant IL-17A (R&D systems) or PBS (control) for different periods of time. To screen the gene expression, genome-wide gene expression analysis was performed on IL-17A stimulated PECs ((10 ng/mL for 24 h) with the Human 12×135K Gene Expression Array (Catalog No. 05543789001) (Roche NimbleGen, Inc., Madison, WI, USA) according to the manufacturer's protocol. To evaluate the role of the MAPK signaling pathway in IL-17A-induced MUC5AC production, specific inhibitors of p38 (SB203580, 5 µM), ERK (U0126, 10 µM) and JNK (SP600125, 25 µM) (all were purchased from Cell Signaling) were used to evaluate the role of the MAPK signaling pathway in IL-17A-induced MUC5AC production. For RNA interference, act1, IL-17RA and IL-17RC siRNA (50 nmol/L) (Genepharma Co., Ltd. Shanghai, China) was transfected into NCI-H292 cells with Lipofectamine 2000 reagent (Invitrogen) according to the manufacturer's protocol. Thereafter, cell pellets and supernatants were collected for further analysis by following the mentioned protocol.

### Statistical analysis

For histological examination, data were expressed as the median and interquartile range (IQR) and were analyzed via the nonparametric Mann-Whitney *U* test. Correlations between the various parameters were assessed by the Spearman rank correlation analysis. For *in vitro* experiments, data were expressed as the means and the standard error of the mean (SEM) and were analyzed with one-way ANOVA and the paired Student's *t* test. A *P* value of less than 0.05 was considered statistically significant.

## Results

**Figure 1:**
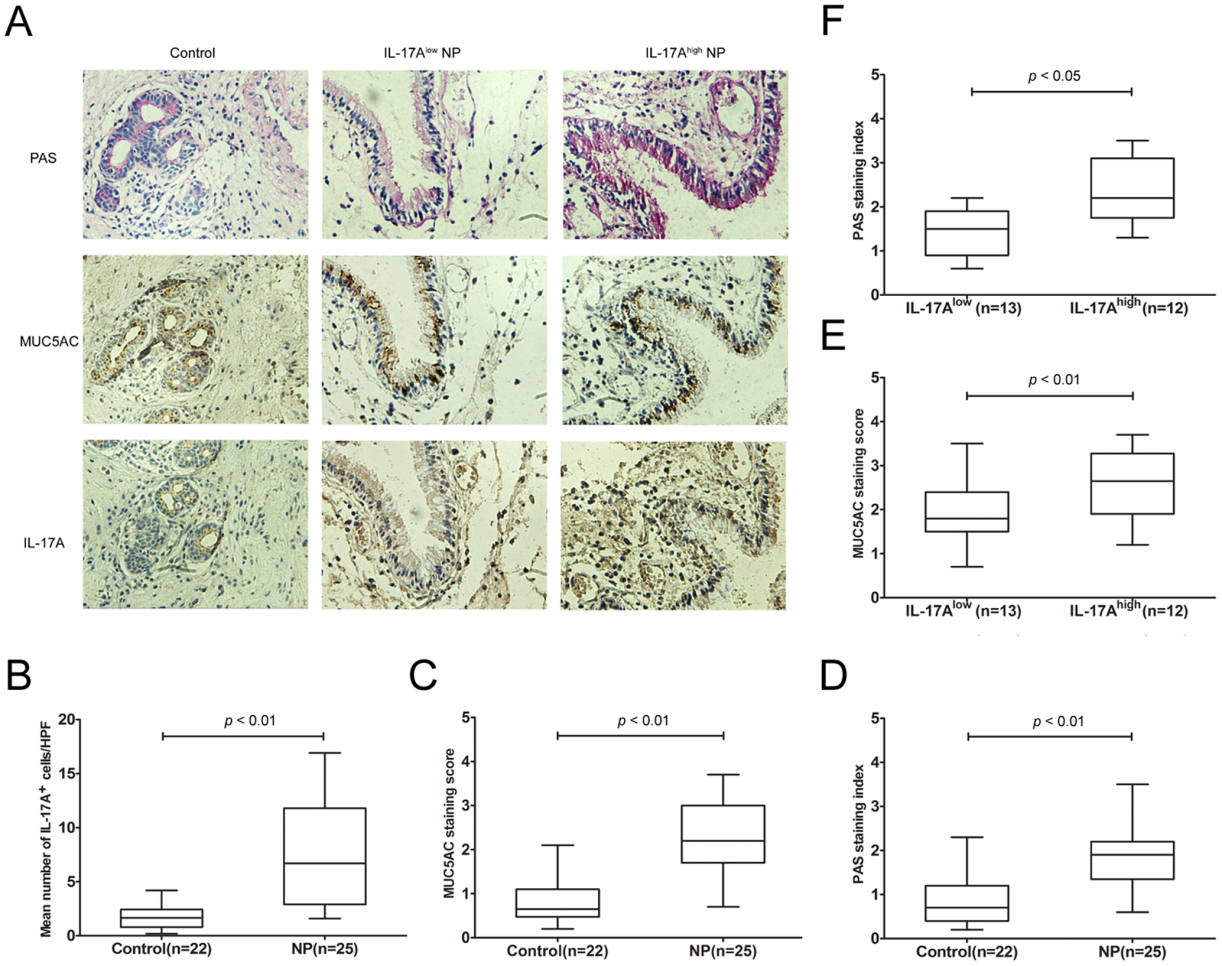
PAS staining and immunostaining of IL-17A and MUC5AC in polyp tissues and normal controls. (A) Representative results of PAS staining and IHC staining of MUC5AC and IL-17A in polyp tissues and normal controls are shown (magnification, 200×). (B) The mean number of IL-17^+^ cells in polyp tissues and normal controls. (C) The PAS staining index in polyp tissues and normal controls. (D) The MUC5AC staining score in polyp tissues and normal controls. (E) The association between IL-17A and the PAS staining index in polyp tissues. (F) The association between IL-17A and MUC5AC staining scores in polyp tissues. Data are expressed as the medians (IQRs).

Because IL-17A has been proposed to be significantly upregulated in nasal polyps, we firstly examined the expression of IL-17A and MUC5AC, as well as goblet cell hyperplasia, in polyp tissues and normal controls. As shown in Fig. 1, the mean number of IL-17A^+^ cells per HPF was 6.7[2.9, 11.8] in polyp tissues and 1.6[0.8, 2.4] in normal controls; the mean staining score of MUC5AC was 2.2[1.7, 3.0] in polyp tissues and 0.6[0.4, 1.1] in normal controls. Both IL-17A and MUC5AC immunostaining were significantly increased in polyp tissues compared with the normal controls (*p*<0.05). Accordingly, we observed more PAS^+^ epithelial cells in polyp tissues, indicating an enhanced goblet cell hyperplasia. The PAS staining index in polyp tissues was 1.9[1.3, 2.2] and was significantly different than in normal controls with a staining index of 0.7[0.4, 1.2] (*p*<0.05). By setting the median of IL-17A^+^ cells in polyp tissues as the cutoff value (6.7/HPFs), we subdivided them into two subgroups: IL-17A^high^ (n = 12) and IL-17^low^ (n = 13). Consequently, the MUC5AC staining score and PAS staining index were significantly higher in IL-17A^high^ subgroup than those in IL-17^low^ subgroup (*p*<0.05). These findings suggested that IL-17A might contribute to MUC5AC expression and goblet cell hyperplasia in polyp tissues.

**Figure 2:**
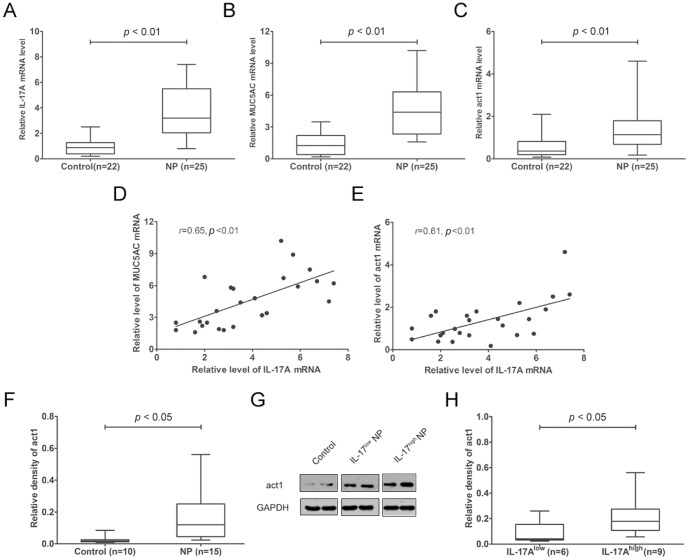
Expression of IL-17A, MUC5AC and act1 in polyp tissues and normal controls. (A–C) The mRNA levels of IL-17A, MUC5AC and act1 in polyp tissues and normal controls. (D) The association of IL-17A and MUC5AC mRNA in polyp tissues. (E) The association of IL-17A and act1 mRNA in polyp tissues. (F) The protein levels of act1 in polyp tissues and normal controls. (G) Representative western blot results of act1 in polyp tissues and normal controls are shown. (H) Densitometric analysis of act1 in polyp tissues and normal controls. The data are expressed as the medians (IQRs).

We next examined the gene expression levels of IL-17A, MUC5AC and act1 in polyp tissues and normal controls. The mRNA level of IL-17A was 3.3[2.0, 5.5] in polyp tissues and 0.8[0.4, 1.2] in normal controls, and the mRNA level of MUC5AC was 4.4[2.3, 6.3] in polyp tissues and 1.2[0.4, 2.2] in normal controls. In accordance with the histological staining, the mRNA levels of IL-17A and MUC5AC were significantly increased in polyp tissues compared with the normal controls (Fig. 2A and B, *p*<0.05). There was significant association of IL-17A and MUC5AC mRNA in polyp tissues (Fig. 2D, *p*<0.05). Moreover, there was significant difference in act1 mRNA in polyp tissues (1.1[0.7, 1.8]) and normal controls (0.8[0.3, 1.2])(Fig 2C, *p*<0.05), and act1 mRNA was significantly correlated with IL-17A mRNA in polyp tissues (Fig 2E, *p*<0.05).

When divided the nasal polyps into IL-17A^high^ and IL-17^low^ subgroup, we found the mRNA levels of IL-17A, MUC5AC and act1 were significantly higher in IL-17A^high^ polyp tissues than those in IL-17A^low^ polyp tissues (*p*<0.05) (Fig S1 in File SI). Correspondingly, act1 protein significantly increased in polyp tissues compared to normal controls (*p*<0.05), and the act1 protein level was significantly higher in the IL-17A^high^ subgroup than those in the IL-17^low^ subgroup (*p*<0.05) (Fig 2F-H). Accordingly, and the protein levels of p-p38, p-EKR and p-JNK were significantly higher in the IL-17A^high^ subgroup than those in the IL-17^low^ subgroup and normal controls (*p*<0.05) (Fig S2 in File SI). These findings suggested that MAPK pathway and act1 might be involved in IL-17A-related MUC5AC expression and goblet cell hyperplasia in polyp tissues.

**Figure 3:**
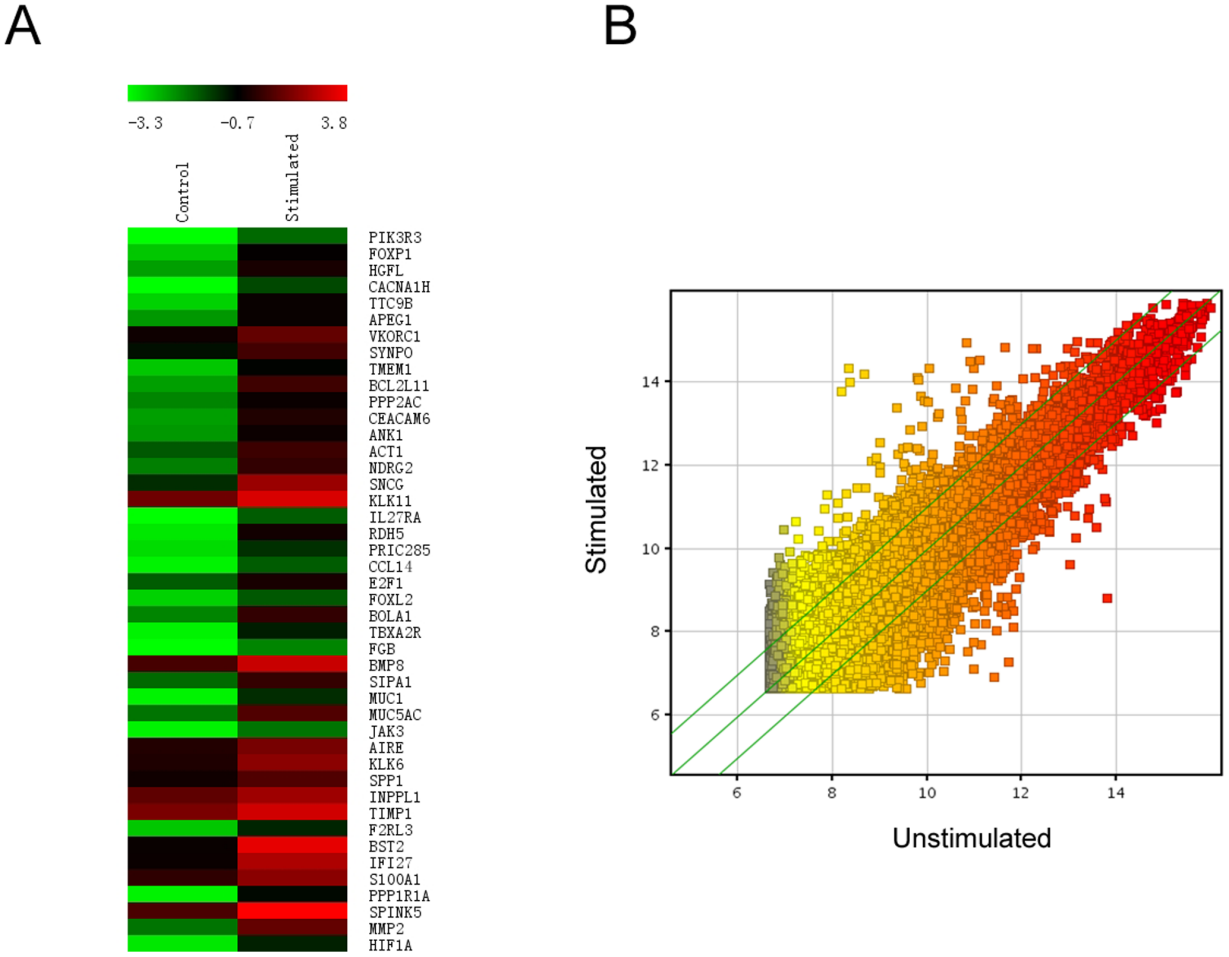
DNA microarray analysis of PECs stimulated by IL-17A. Shown are the heatmap of genes upregulated in stimulated PECs (A) and the scatter-plot for stimulated *vs* unstimulated PECs (B).

**Figure 4:**
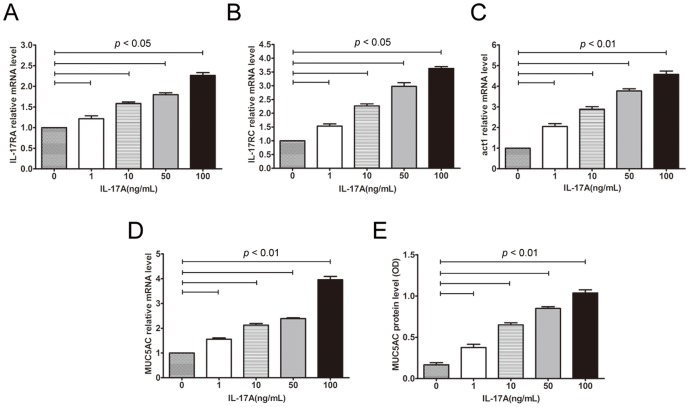
IL-17A induced IL-17RA, IL-17RC, act1 and MUC5AC expression in PECs and NCI-H292 cells *in vitro*. (A-D) The IL-17RA, IL-17RC, act1 and MUC5AC mRNA levels in PECs after IL-17A (0–100 ng/mL) stimulation for 12 h. (E) The MUC5AC protein level in PECs after IL-17A (0–100 ng/mL) stimulation for 24 h. The data are expressed as the means (SEM) of 3 independent experiments.

To assess the regulatory effect of IL-17A on gene expression in nasal epithelial cells *in vitro*, we then screened the upregulated gene expression in cultured PECs by DNA microarray analysis after IL-17A stimulation (10 ng/mL) for 24 h. As a result, we identified 46 upregulated related genes (Fig 3). Of these genes, MUC5AC was increased 6.25 fold in stimulated PECs, and act1 was increased 4 fold in PECs. To further validate the promotive effect of IL-17A on MUC5AC expression and the underlying pathways, we examined IL-17RA, IL-17RC, act1 and MUC5AC mRNA expression after IL-17A stimulation. As shown in Fig. 4, IL-17A significantly increased IL-17RA, IL-17RC, act1 and MUC5AC mRNA expression in cultured PECs in a dose-dependent manner (*p*<0.05). Accordingly, MUC5AC protein level was dose-dependently upregulated in cultured PECs in the presence of IL-17A as well (*p*<0.05).

**Figure 5:**
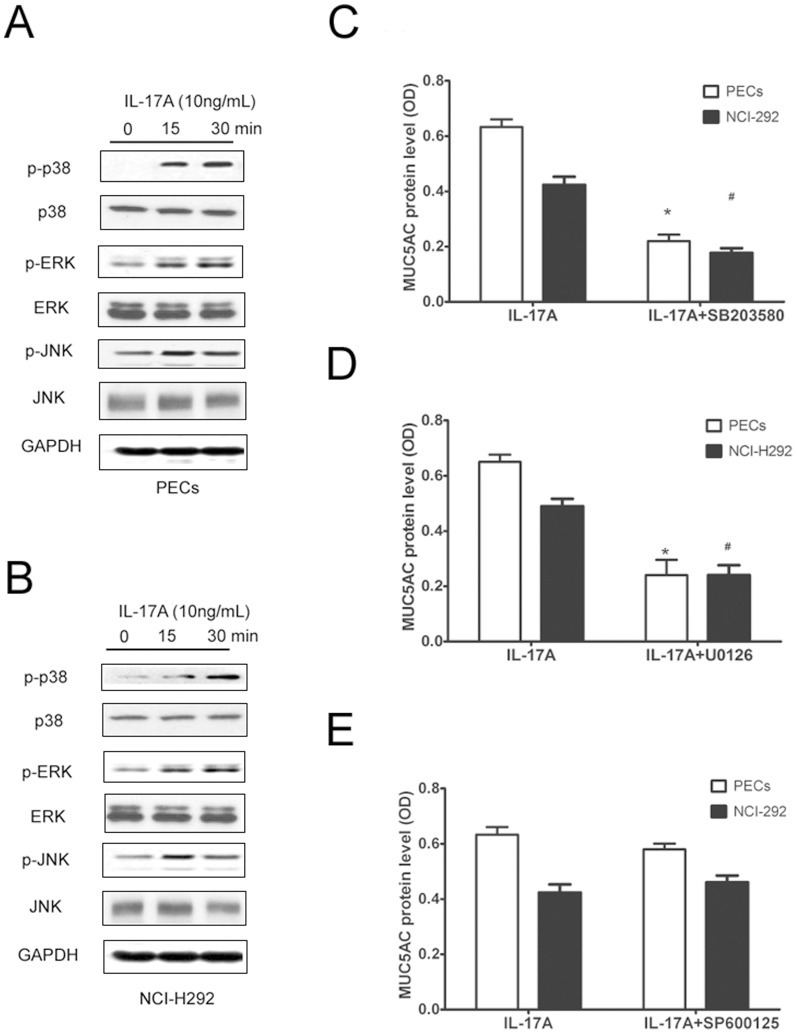
MAPK signaling mediated IL-17A induced MUC5AC in PECs and NCI-H292 cells *in vitro*. (A) Representative western blot result of phosphorylated p38, ERK and JNK in PECs after IL-17A stimulation. (B) Representative western blot result of phosphorylated p38, ERK and JNK in NCI-H292 cells after IL-17A stimulation. (C, D) MUC5AC protein level in cultured PECs and NCI-H292 cells after IL-17A stimulation for 24 h in the presence of specific inhibitors of p38, ERK and JNK. The data are expressed the means (SEM) of 3 independent experiments. * *p*<0.05 when compared with control PECs, ^#^ *p*<0.05 when compared with control NCI-H292 cells.

**Figure 6:**
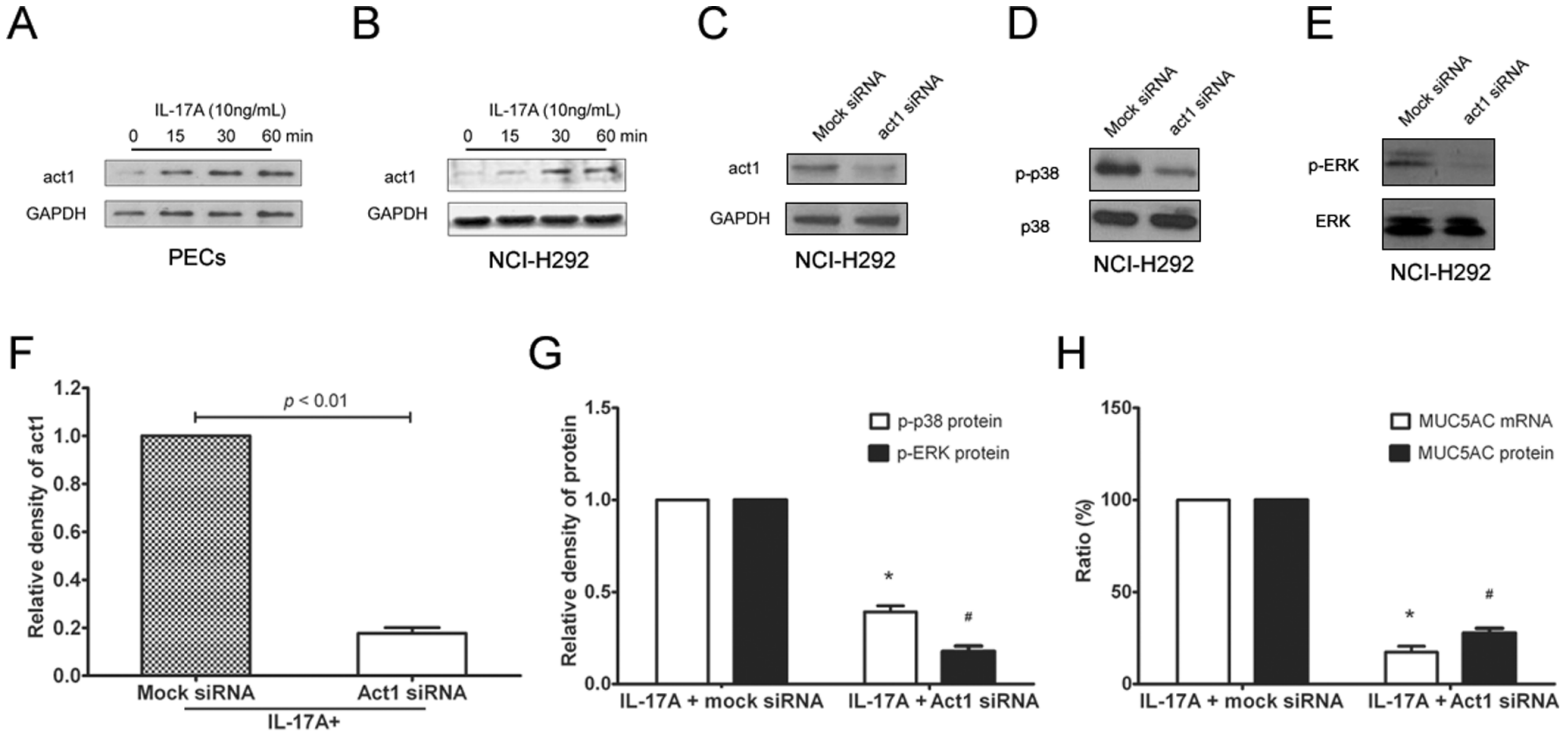
Act1 is required for IL-17A-induced MUC5AC expression *in vitro*. (A, B) Representative result of western blots for act1 in PECs and NCI-H292 cells after IL-17A stimulation. (C–E) Representative result of western blots for act1, p-p38 and p-ERK in NCI-H292 cells in the presence of IL-17A and/or act1 siRNA. (F) Relative level of act1 in NCI-H292 cells in the presence of IL-17A and act1 siRNA. (G, H) Relative level of p-p38 and p-ERK in NCI-H292 cells in the presence of IL-17A and act1 siRNA. (E) Relative levels of MUC5AC mRNA and protein in NCI-H292 cells in the presence of IL-17A and act1 siRNA. The data indicated the means (SEM) of 3 independent experiments. * *p*<0.05 when compared with control PECs, ^#^ *p*<0.05 when compared with control NCI-H292 cells.

We next examined the importance of the MAPK pathway in IL-17A-induced MUC5AC expression in cultured PECs and NCI-H292 cells. Based on the preliminary experiment, we used 10 ng/mL of IL-17A as the optimal concentration for stimulation. As shown in Fig. 5A and B, IL-17A significantly increased p-p38, p-ERK and p-JNK in both PECs and NCI-H292 cells in a time-dependent manner. When adding specific inhibitors of p-p38 (SB203580), p-ERK (U0126) and p-JNK (SP600125), we found SB203580 and U0126, but not SP600125, significantly inhibited IL-17A-induced MUC5AC production (Fig. 5C–E, *p*<0.05), suggesting activated p38 and ERK were involved in MUC5AC expression in response to IL-17A stimulation. To further evaluate the importance of IL-17RA, IL-17RC and act1 in IL-17A-induced MUC5AC expression *in vitro*, we examined IL-17A-induced MUC5AC production in cultured NCI-H292 in the presence of IL-17RA, IL-17RC and act1 siRNA. Consequently, we found both IL-17RA and IL-17RC siRNA significantly inhibited the mRNA and protein levels of MUC5AC in IL-17A induced NCI-H292 cells (Fig S3 in File SI). As to act1 expression, IL-17A stimulation significantly increased act1 protein expression in cultured PECs and NCI-H292 cells in a time-dependent manner (Fig. 6A and B, *p*<0.05). When adding act1 siRNA, we found that act1, p-p38 and p-ERK protein levels were significantly inhibited in NCI-H292 cells compared with the control group (Fig. 6C–G, *p*<0.05). Consistently, MUC5AC mRNA and protein levels were significantly inhibited in NCI-H292 cells compared with the control group (Fig 6H, *p*<0.05). These findings suggested that IL-17RA and IL-17RC and act1 were required for IL-17A-induced MUC5AC production in airway epithelial cells.

## Discussion

In the present study, we have demonstrated enhanced MUC5AC expression and goblet cell hyperplasia in NP patients, which were correlated with IL-17A levels. Furthermore, we have shown that IL-17A significantly increases MUC5AC production in cultured airway epithelial cells, and provide evidence that act1-mediated signaling was required for IL-17A-induced MUC5AC production by using siRNA interference. Therefore, our finding may be crucial for the understanding of pathophysiology of NP patients and contribute to the establishment of optimal therapeutic strategy.

Profound mucus accumulation and goblet cell hyperplasia in the airway are commonly observed in NP patients [17]. These abnormalities in goblet cell number and stored mucins, such as MUC5AC, contribute to subsequent nasal congestion and rhinorrhea. There are numerous publications concerning MUC5AC expression in NP patients [18], [19]. In agreement with the previous studies, we found that MUC5AC expression was significantly increased in NP patients. The molecular mechanisms underlying excessive MUC5AC production in chronic airway diseases are not fully understood; thus, the optimal therapeutic intervention for mucin remains controversial [20]–[22]. Previously, it was proposed that airway goblet cell hyperplasia and profound mucus hypersecretion are largely driven by the Th2 cytokine IL-13 [13], [15]. For example, IL-13 has been demonstrated to induce goblet cell hyperplasia in human airway epithelial cells in vitro [13], and blocking IL-13 significantly inhibits mucus overproduction in a murine model of asthma [15]. However, when we attempted to evaluate the importance of IL-13 in MUC5AC production and glandular hyperplasia, we only observed very low levels of IL-13 in these polyp tissues and we failed to establish association between IL-13 and MUC5AC expression in this cohort (data not shown). These made us to look for other candidate drivers for mucin hypersecretion in Chinese NP patients.

Due to the absence or low expression of IL-17A in polyp tissues in western patients [23], Th17 cells have not been well documented in the pathogenesis of NP. Recently, some studies have demonstrated that Th17 cells are specifically increased in NP patients in China and eastern Asian regions compared to their western counterparts [16], [24]. Increased IL-17A has been proposed to be responsible for enhanced tissue neutrophilia, collagen deposition and corticosteroid resistance in polyp tissues [10], [25], [26]. In this study, we established a close relationship between excessive MUC5AC expression, goblet cell hyperplasia and IL-17A production in polyp tissues, providing the clue that Th17 cells may play an important role in the excessive MUC5AC production and goblet cell hyperplasia.

IL-17A, produced by Th17 cells, is the prototypic IL-17 family member. IL-17A can cause accumulation of neutrophils in the bronchoalveolar lavage fluid of rats and mice *in vivo*. The main function of IL-17A is to coordinate local tissue inflammation via the up-regulation of proinflammatory and neutrophil-mobilizing cytokines and chemokines (including IL-6, G-CSF, TNF-α, IL-1β, CCL2 and CCL20), as well as matrix metalloproteases, to allow activated T cells to penetrate the extracellular matrix [27]. Recent studies have demonstrated that the Th17 cytokine IL-17A is able to promote airway goblet cell hyperplasia and profound mucus hypersecretion through an NF-κB-based transcriptional mechanism [14]. Because IL-17A has also been demonstrated to modulate corticosteroid sensitivity by increasing the expression of corticosteroid β receptor [28], it is reasonable to speculate that IL-17A-induced MUC5AC production and goblet cell hyperplasia were specifically resistant to corticosteroid therapy in chronic airway diseases. Therefore, it demands a further understanding of the signaling pathway underlying IL-17A-induced MUC5AC production to establish an available therapeutic strategy.

IL-17A signals through a heteromeric receptor complex, consisting of IL-17R (IL-17RA) and IL-17RC, which are single-pass transmembrane proteins and ubiquitously expressed in various cell types including epithelial cells and fibroblasts [29]. Recently, a novel signaling molecule, act1, was found to be a key component in IL-17A signaling [30]. The act1 gene was first cloned as an NF-κB activator. It contains two TNF receptor-associated factor binding sites and a helix-loop-helix domain at the N terminus [31]. Act1 deficiency in epithelial cells caused loss of IL-17-induced neutrophilia and reduced the phenotype of allergic pulmonary inflammation, indicating that the act1-mediated signaling is crucial for IL-17A-induced inflammatory response [32]. In the present study, we found that act1 expression was significantly increased in polyp tissues compared with normal controls. The increased act1 production was shown to be significantly associated with IL-17A levels in polyp tissues, providing a clue that the IL-17A/act1 axis may play a crucial role in MUC5AC production in NP patients.

How act1 contributes to IL-17A-mediated molecular events has not been fully understood. Some authors proposed that act1 engages IL-17RA and IL-17RC through SEFIR–SEFIR domain interactions following IL-17 stimulation. This process is followed by the recruitment of TNF receptor-associated factor-6 and transforming growth factor β-activated kinase 1 kinase, which mediates the downstream activation of NF-κB [33]. Importantly, a recent study has indicated that act1 is a confirmed E3 ubiquitin ligase through its U-box-like region. Activity of E3 ubiquitin ligase is essential for IL-17-mediated signaling pathways and inflammatory gene expression [31]. However, whether act1 participates in IL-17A-induced MUC5AC production in airway epithelial cells remains unclear. To address this issue, we examined IL-17A-induced changes of gene expression *in vitro*. By using DNA microarray analysis, we identified 46 upregulated related genes, including MUC5AC (6.25 fold) and act1 (4 fold), in nasal epithelial cells, confirming the association of IL-17A, MUC5AC and act1 in polyp tissues. Other inflammation-related genes such as MMP2 and HIF1A were shown to be significantly upregulated in response to IL-17A, which may need further investigation. Correspondingly, we found that IL-17A significantly increased IL-17RA, IL-17RC, act1 and MUC5AC mRNA expression in cultured PECs in a dose-dependent manner. These findings support the promotive effect of IL-17A on MUC5AC expression and possible role of the act1-mediated pathway in MUC5AC production. In addition, we examined the importance of the MAPK pathway, IL-17RA, IL-17RC and act1 in IL-17A-induced MUC5AC expression by using specific inhibitors and siRNA. Consequently, our results showed that IL-17RA, IL-17RC, act1, as well as p38- and ERK-mediated pathways, were involved in MUC5AC expression in response to IL-17A stimulation. This newly identified mechanism suggests that act1 may represent a promising target for treating goblet cell proliferation, differentiation and mucus hypersecretion in Th17-dominant NP patients [34].

## Conclusion

In summary, we have demonstrated that MUC5AC expression and goblet cell hyperplasia were significantly enhanced in NP patients, which were associated with act1-mediated signaling. This newly identified mechanism suggests that act1-mediated signaling may represent a promising target for treating goblet cell proliferation, differentiation and mucus hypersecretion in Th17-dominant NP patients.

## Supporting Information

File S1 Contains the following files: Table S1. Descriptive characteristics of NPC patients and normal controls. Table S2. Sequences of primers for qPCR analysis. Fig. S1. The mRNA levels of IL-17A, MUC5AC and act1 in IL-17A^high^ and IL-17A^low^ polyp tissues. Fig. S2. The levels of p-p38, p-ERK, p-JNK protein in IL-17A^high^ and IL-17A^low^ polyp tissues and normal controls, as suggested by western blot analysis. Fig. S3. The mRNA and protein levels of MUC5AC in IL-17A induced NCI-H292 cells, in the presence or absence of IL-17RA and IL-17RC siRNA.
(DOC)

## References

[1] Fokkens WJ, Lund VJ, Mullol J, Bachert C, Alobid I, et al (2012) EPOS 2012: European position paper on rhinosinusitis and nasal polyps 2012. A summary for otorhinolaryngologists. Rhinology 50: 1–12. 22469599 10.4193/Rhino12.000

[2] Hsu J, Peters AT (2011) Pathophysiology of chronic rhinosinusitis with nasal polyp. Am J Rhinol Allergy 25: 285–90. 22186239 10.2500/ajra.2011.25.3680

[3] Kim JW, Hong SL, Kim YK, Lee CH, Min YG, et al (2007) Histological and immunological features of non-eosinophilic nasal polyps. Otolaryngol Head Neck Surg 137: 925–30. 18036422 10.1016/j.otohns.2007.07.036

[4] Cao PP, Li HB, Wang BF, Wang SB, You XJ, et al (2009) Distinct immunopathologic characteristics of various types of chronic rhinosinusitis in adult Chinese. J Allergy Clin Immunol 124: 478–84. 19541359 10.1016/j.jaci.2009.05.017

[5] Shi LL, Xiong P, Zhang L, Cao PP, Liao B, et al (2013) Features of airway remodeling in different types of Chinese chronic rhinosinusitis are associated with inflammation patterns. Allergy 68: 101–9. 23157215 10.1111/all.12064

[6] Halwani R, Al-Muhsen S, Hamid Q (2013) T helper 17 cells in airway diseases: from laboratory bench to bedside. Chest 143: 494–501. 23381314 10.1378/chest.12-0598

[7] Miossec P, Kolls JK (2012) Targeting IL-17 and TH17 cells in chronic inflammation. Nat Rev Drug Discov 11: 763–76. 23023676 10.1038/nrd3794

[8] Hartupee J, Liu C, Novotny M, Li X, Hamilton T (2007) IL-17 enhances chemokine gene expression through mRNA stabilization. J Immunol 179: 4135–41. 17785852 10.4049/jimmunol.179.6.4135

[9] Datta S, Novotny M, Pavicic PG Jr, Zhao C, Herjan T, et al (2010) IL-17 regulates CXCL1 mRNA stability via an AUUUA/tristetraprolin-independent sequence. J Immunol 184: 1484–91. 20042592 10.4049/jimmunol.0902423 PMC2829999

[10] Derycke L, Zhang N, Holtappels G, Dutré T, Bachert C (2012) IL-17A as a regulator of neutrophil survival in nasal polyp disease of patients with and without cystic fibrosis. J Cyst Fibros 11: 193–200. 22178076 10.1016/j.jcf.2011.11.007

[11] Chen Y, Thai P, Zhao YH, Ho YS, DeSouza MM, et al (2003) Stimulation of airway mucin gene expression by interleukin (IL)-17 through IL-6 paracrine/autocrine loop. J Biol Chem 278: 17036–43. 12624114 10.1074/jbc.M210429200

[12] Curran DR, Cohn L (2010) Advances in mucous cell metaplasia: a plug for mucus as a therapeutic focus in chronic airway disease. Am J Respir Cell Mol Biol 42: 268–75. 19520914 10.1165/rcmb.2009-0151TR PMC2830403

[13] Kanoh S, Tanabe T, Rubin BK (2011) IL-13-induced MUC5AC production and goblet cell differentiation is steroid resistant in human airway cells. Clin Exp Allergy 41: 1747–56. 22092504 10.1111/j.1365-2222.2011.03852.x

[14] Fujisawa T, Velichko S, Thai P, Hung LY, Huang F, et al (2009) Regulation of airway MUC5AC expression by IL-1beta and IL-17A; the NF-kappaB paradigm. J Immunol 183: 6236–43. 19841186 10.4049/jimmunol.0900614 PMC4623590

[15] Alevy YG, Patel AC, Romero AG, Patel DA, Tucker J, et al (2012) IL-13-induced airway mucus production is attenuated by MAPK13 inhibition. J Clin Invest 122: 4555–68. 23187130 10.1172/JCI64896 PMC3533556

[16] Wang H, Bai J, Ding M, Liu W, Xu R, et al (2013) Interleukin-17A contributes to the expression of serum amyloid A in chronic rhinosinusitis with nasal polyps. Eur Arch Otorhinolaryngol 270: 1867–72. 23247332 10.1007/s00405-012-2295-x

[17] Van Crombruggen K, Zhang N, Gevaert P, Tomassen P, Bachert C (2011) Pathogenesis of chronic rhinosinusitis: inflammation. J Allergy Clin Immunol 128: 728–32. 21868076 10.1016/j.jaci.2011.07.049

[18] Ali MS, Wilson JA, Bennett M, Pearson JP (2005) Mucin gene expression in nasal polyps. Acta Otolaryngol 125: 618–24. 16076710 10.1080/00016480510027538

[19] Kim CH, Song KS, Kim SS, Kim HU, Seong JK, et al (2000) Expression of MUC5AC mRNA in the goblet cells of human nasal mucosa. Laryngoscope 110: 2110–3. 11129031 10.1097/00005537-200012000-00026

[20] Martínez-Antón A, de Bolós C, Alobid I, Benítez P, Roca-Ferrer J, et al (2008) Corticosteroid therapy increases membrane-tethered while decreases secreted mucin expression in nasal polyps. Allergy 63: 1368–76. 18547287 10.1111/j.1398-9995.2008.01678.x

[21] Burgel PR, Cardell LO, Ueki IF, Nadel JA (2004) Intranasal steroids decrease eosinophils but not mucin expression in nasal polyps. Eur Respir J 24: 594–600. 15459138 10.1183/09031936.04.00014404

[22] Tanabe T, Kanoh S, Tsushima K, Yamazaki Y, Kubo K, et al (2011) Clarithromycin inhibits interleukin-13-induced goblet cell hyperplasia in human airway cells. Am J Respir Cell Mol Biol 45: 1075–83. 21642590 10.1165/rcmb.2010-0327OC

[23] Peters AT, Kato A, Zhang N, Conley DB, Suh L, et al (2010) Evidence for altered activity of the IL-6 pathway in chronic rhinosinusitis with nasal polyps. J Allergy Clin Immunol 125: 397–403. 20159251 10.1016/j.jaci.2009.10.072 PMC2828355

[24] Hu XD, Bao YY, Zhou SH, Yao HT, Mao JY, et al (2013) Interleukin-17A expression in patients with chronic rhinosinusitis and its relationship with clinical features. J Int Med Res 41: 777–84. 23613503 10.1177/0300060513478089

[25] Molet SM, Hamid QA, Hamilos DL (2003) IL-11 and IL-17 expression in nasal polyps: relationship to collagen deposition and suppression by intranasal fluticasone propionate. Laryngoscope 113: 1803–12. 14520110 10.1097/00005537-200310000-00027

[26] Zijlstra GJ, Ten Hacken NH, Hoffmann RF, van Oosterhout AJ, Heijink IH (2012) Interleukin-17A induces glucocorticoid insensitivity in human bronchial epithelial cells. Eur Respir J 39: 439–45. 21828034 10.1183/09031936.00017911

[27] Fouser LA, Wright JF, Dunussi-Joannopoulos K, Collins M (2008) Th17 cytokines and their emerging roles in inflammation and autoimmunity. Immunol Rev 226: 87–102. 19161418 10.1111/j.1600-065X.2008.00712.x

[28] Vazquez-Tello A, Semlali A, Chakir J, Martin JG, Leung DY, et al (2010) Induction of glucocorticoid receptor-beta expression in epithelial cells of asthmatic airways by T-helper type 17 cytokines. Clin Exp Allergy 40: 1312–22. 20545708 10.1111/j.1365-2222.2010.03544.x

[29] Gaffen SL (2009) Structure and signalling in the IL-17 receptor family. Nat Rev Immunol 9: 556–67. 19575028 10.1038/nri2586 PMC2821718

[30] Qian Y, Liu C, Hartupee J, Altuntas CZ, Gulen MF, et al (2007) The adaptor Act1 is required for interleukin 17-dependent signaling associated with autoimmune and inflammatory disease. Nat Immunol 8: 247–56. 17277779 10.1038/ni1439

[31] Liu C, Qian W, Qian Y, Giltiay NV, Lu Y, et al (2009) Act1, a U-box E3 ubiquitin ligase for IL-17 signaling. Sci Signal 2: ra63. 19825828 10.1126/scisignal.2000382 PMC3182834

[32] Swaidani S, Bulek K, Kang Z, Liu C, Lu Y, et al (2009) The critical role of epithelial-derived Act1 in IL-17- and IL-25-mediated pulmonary inflammation. J Immunol 182: 1631–40. 19155512 10.4049/jimmunol.182.3.1631 PMC3015148

[33] Shi P, Zhu S, Lin Y, Liu Y, Liu Y, et al (2011) Persistent stimulation with interleukin-17 desensitizes cells through SCFβ-TrCP-mediated degradation of Act1. Sci Signal 4: ra73. 22045853 10.1126/scisignal.2001653

[34] Turner J, Jones CE (2009) Regulation of mucin expression in respiratory diseases. Biochem Soc Trans 37: 877–81. 19614611 10.1042/BST0370877

