# Supplementary material for: Interleukin-17A Promotes MUC5AC Expression and Goblet Cell Hyperplasia in Nasal Polyps via the Act1-Mediated Pathway
Source: PLoS One. 2014 Jun 3;9(6):e98915. doi: 10.1371/journal.pone.0098915 (PMC4043856; doi:10.1371/journal.pone.0098915)
Supplement: File S1 — Contains the following files: Table S1. Descriptive characteristics of NPC patients and normal controls. Table S2. Sequences of primers for qPCR analysis. Fig. S1. The mRNA levels of IL-17A, MUC5AC and act1 in IL-17Ahigh and IL-17Alow polyp tissues. Fig. S2. The levels of p-p38, p-ERK, p-JNK protein in IL-17Ahigh and IL-17Alow polyp tissues and normal controls, as suggested by western blot analysis. Fig. S3. The mRNA and protein levels of MUC5AC in IL-17A induced NCI-H292 cells, in the presence or absence of IL-17RA and IL-17RC siRNA. (DOC) [file pone.0098915.s001.doc]

**Supporting Information**

**Table S1**. Descriptive characteristics of NPC patients and normal controls

**Table S2**. Sequences of primers for qPCR analysis

**Fig. S1.** The mRNA levels of IL-17A, MUC5AC and act1 in IL-17A^high^ and IL-17A^low^ polyp tissues.

**Fig. S2.** The levels of p-p38, p-ERK, p-JNK protein in IL-17A^high^ and IL-17A^low^ polyp tissues and normal controls, as suggested by western blot analysis.

**Fig. S3.** The mRNA and protein levels of MUC5AC in IL-17A induced NCI-H292 cells, in the presence or absence of IL-17RA and IL-17RC siRNA.

**Table S1**. Descriptive characteristics of NPC patients and normal controls

| Subject | Healthy controls | NP patients |
| --- | --- | --- |
| No. of patients | 22 | 25 |
| Gender |  |  |
| Males/Females | 12/10 | 14/11 |
| Age (yrs) | 32 (22-54) | 36 (25-60) |
| Duration (yrs) | - | 4 (2-7) |
| Skin prick test |  |  |
| Positive/Negative | - | 9/16 |
| Asthma history |  |  |
| Yes/No | - | 4/21 |
| Smoking |  |  |
| Yes/No | - | 7/18 |

Age and duration: median (range).

**Table S2**. Sequences of primers for qPCR analysis

| Primer | Sequence |
| --- | --- |
| IL-17A | Forward: 5’- CAA CCG ATC CAC CTC ACC-3’ |
|  | Reverse: 5’- AGC CCA CGG ACA CCA GTA-3’ |
| MUC5AC | Forward: 5’- TGC GTC CCA CGA CAT CTG -3’ |
|  | Reverse: 5’- CAG GTG AAT GGG CAC ATG TG -3’ |
| IL-17RA | Forward: 5’- CAG ACA GAC GCC AGC ATC CT -3’ |
|  | Reverse: 5’- GTG ATG CCT CAG TTT GGA CAG A -3’ |
| IL-17RC | Forward: 5’- TGT CTG CAG CTA TGG GAC GA -3’ |
|  | Reverse: 5’- GCA AAG AGT AGG CAG GCC AG -3’ |
| Act1 | Forward: 5’- gca ttc ctg tgg agg ttg at -3’ |
|  | Reverse: 5’- gtc tcc gga gga att gtg aa -3’ |
| GAPDH | Forward: 5’- GAA GGT GAA GGT CGG AGT -3’ |
|  | Reverse: 5’- GAA GAT GGT GAT GGG ATT TC-3’ |

**
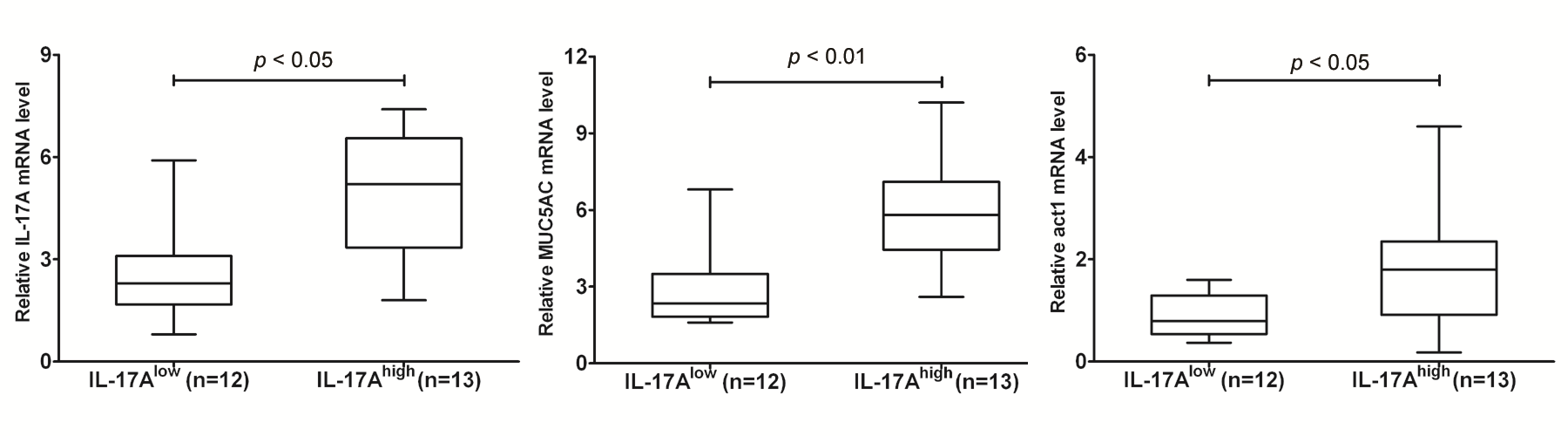
Fig. S1.** The mRNA levels of IL-17A, MUC5AC and act1 in IL-17A^high^ and IL-17A^low^ polyp tissues. (A) The mRNA level of IL-17A in IL-17A^high^ and IL-17A^low^ polyp tissues. (B) The mRNA level of MUC5AC in IL-17A^high^ and IL-17A^low^ polyp tissues. (C) The mRNA level of act1 in IL-17A^high^ and IL-17A^low^ polyp tissues. Data are expressed as the medians (IQRs).

**
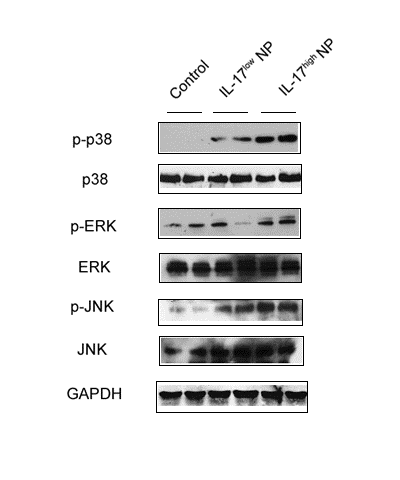
**

**Fig. S2.** The levels of p-p38, p-ERK, p-JNK protein in IL-17A^high^ and IL-17A^low^ polyp tissues and normal controls, as suggested by western blot analysis. Representative results of western blot analysis were shown.

**
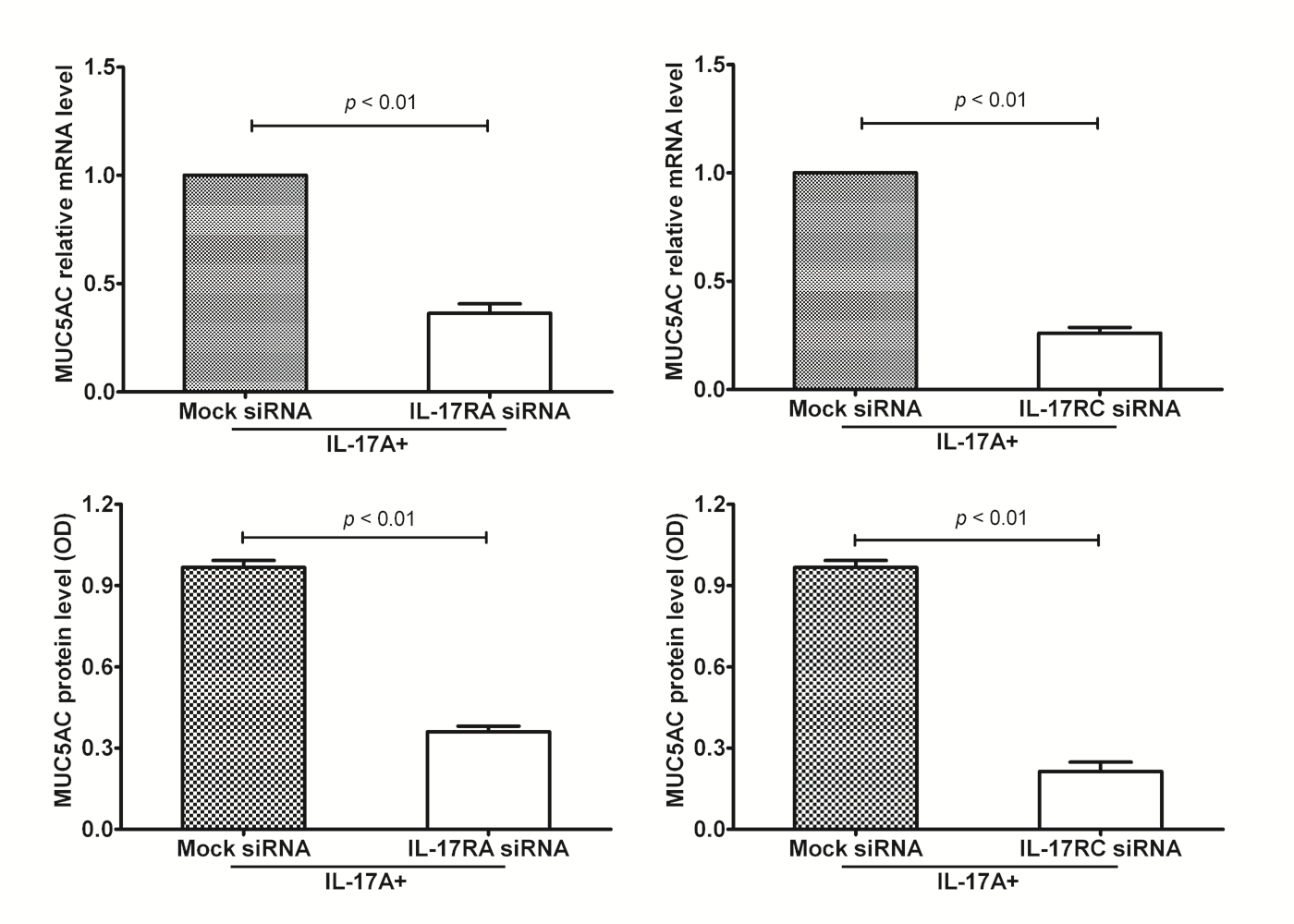
**

**Fig. S3.** The mRNA and protein levels of MUC5AC in IL-17A induced NCI-H292 cells, in the presence or absence of IL-17ARA and IL-17RC siRNA. Both IL-17RA and IL-17RC siRNA significantly inhibited the mRNA and protein levels of MUC5AC in IL-17A induced NCI-H292 cells. The MUC5AC mRNA and protein levels were shown in NCI-H292 cells after IL-17A (10 ng/mL) stimulation for 12 or 24 h. The data are expressed as the means (SEM) of 3 independent experiments.
